# Stimulation of soluble guanylyl cyclase protects against obesity by recruiting brown adipose tissue

**DOI:** 10.1038/ncomms8235

**Published:** 2015-05-26

**Authors:** Linda S. Hoffmann, Jennifer Etzrodt, Lena Willkomm, Abhishek Sanyal, Ludger Scheja, Alexander W.C. Fischer, Johannes-Peter Stasch, Wilhelm Bloch, Andreas Friebe, Joerg Heeren, Alexander Pfeifer

**Affiliations:** 1 Institute of Pharmacology and Toxicology, University Hospital Bonn, University of Bonn, Bonn D-53105, Germany; 2 Department of Molecular and Cellular Sports Medicine, German Sport University Cologne, Institute of Cardiovascular Research and Sports Medicine, Cologne D-50735, Germany; 3 Research Training Group 1873, University of Bonn, Bonn D-53127, Germany; 4 Department of Biochemistry and Molecular Cell Biology, University Medical Center Hamburg-Eppendorf, Hamburg D-20246, Germany; 5 Bayer Pharma AG, Wuppertal D-42113, Germany; 6 Institute of Pharmacy, Martin Luther University Halle-Wittenberg, Halle an der Saale D-06120, Germany; 7 Institute of Physiology, Martin Luther University Würzburg, Würzburg D-97070, Germany; 8 PharmaCenter, University of Bonn, Bonn D-53119, Germany

## Abstract

Obesity is characterized by a positive energy balance and expansion of white adipose tissue (WAT). In contrast, brown adipose tissue (BAT) combusts energy to produce heat. Here we show that a small molecule stimulator (BAY 41-8543) of soluble guanylyl cyclase (sGC), which produces the second messenger cyclic GMP (cGMP), protects against diet-induced weight gain, induces weight loss in established obesity, and also improves the diabetic phenotype. Mechanistically, the haeme-dependent sGC stimulator BAY 41–8543 enhances lipid uptake into BAT and increases whole-body energy expenditure, whereas ablation of the haeme-containing β_1_-subunit of sGC severely impairs BAT function. Notably, the sGC stimulator enhances differentiation of human brown adipocytes as well as induces ‘browning' of primary white adipocytes. Taken together, our data suggest that sGC is a potential pharmacological target for the treatment of obesity and its comorbidities.

## Abstract (web summary)

The enzyme soluble guanylyl cyclase (sGC) regulates differentiation of brown fat. Here, Hoffman *et al.* show that a small molecule sGC stimulator increases brown fat activity and browning of white fat, thereby inducing energy expenditure, weight loss and partial protection from diet-induced obesity in mice.

## Graphical abstract

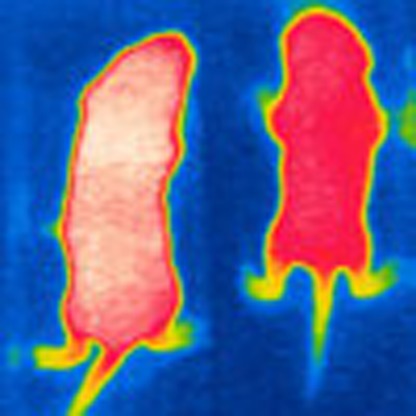

Obesity is a worldwide health problem and has reached pandemic dimensions^1^. When energy intake constantly exceeds energy expenditure (EE), the surplus is stored as lipids in white adipose tissue (WAT) leading to the development of obesity. Obesity is associated with comorbidities such as type 2 diabetes, metabolic syndrome, cardiovascular diseases and cancer^2^. WAT not only serves as the major energy storage but also has endocrine functions. It secretes adipokines such as leptin or tumour-necrosis factor-α, which can affect metabolism and can enhance inflammation^3^. In contrast to WAT, brown adipose tissue (BAT) dissipates energy and generates heat by non-shivering thermogenesis. BAT-dependent energy dissipation plays a central role in the defense against cold, a process that is dependent on mitochondrial energy dissipation mediated by uncoupling protein 1 (UCP1)^4^,^5^,^6^,^7^,^8^,^9^,^10^.

BAT is subjected to an intriguing level of plasticity and the total number of brown adipocytes (BA) in the body can vary extensively. In humans, BAT diminishes with age^11^ and increased body weight^12^—a process also known as ‘whitening'^13^. On the other hand, cold exposure and several other stimuli including cGMP signalling^14^ can induce browning, the appearance of inducible BA in WAT^15^,^16^,^17^,^18^—so-called beige or brite cells. Inducible BA share several characteristic features with classical BAT including multilocular lipid droplets, high mitochondria content and expression of UCP1, which allows them to consume energy like classical BA^10^,^16^.

Cyclic nucleotides play an important role in the control of adipocyte function^4^,^7^,^9^,^10^ and therefore have a high potential to be exploited in antiobesity therapies. The major focus of BAT research has been on cAMP, which is a key inducer of thermogenesis. Cold exposure results in the release of norepinephrine (NE) from sympathetic nerves in BAT and subsequent activation of β-adrenergic receptors that couple via G_s_ protein to adenylate cylcase, thereby stimulating cAMP production. Moreover, it has been shown recently that adenosine is a sympathetic co-transmitter that induces cAMP production via A_2A_ receptors in BAT^19^. cAMP-initiated lipolysis results in the release of free fatty acids and activation of UCP1, which disrupts the mitochondrial proton gradient and causes the production of heat instead of ATP^4^.

Apart from cAMP, the other cyclic nucleotide cyclic GMP (cGMP) also plays an important role in BAT^20^,^21^. cGMP is produced by soluble and membrane-bound guanylyl cyclases that are activated by NO and natriuretic peptides, respectively^22^. cGMP-dependent protein kinase GI (PKGI) is the major downstream mediator of cGMP actions and has been shown to be indispensible for BAT thermogenesis and crucially involved in browning of WAT^20^,^23^. Very recently, it was demonstrated that dietary nitrate, a NO-releasing compound, can induce browning *in vitro* and *in vivo*^24^. Natriuretic peptides such as ANP also play an important role in browning of white adipocytes and can increase EE on the cellular and whole-body level^25^.

Given the high medical need for new antiobesity therapies, we focused on BAT-centred therapies based on the soluble guanylyl cyclase (sGC)-dependent pathway, which might be used to regulate BAT plasticity to increase BAT mass. We found that a pharmacological stimulator of sGC counteracts diet-induced obesity (DIO) and results in favourable metabolic changes such as improved glucose tolerance.

## Results

### sGC deficiency impairs BA function

**Figure 1:**
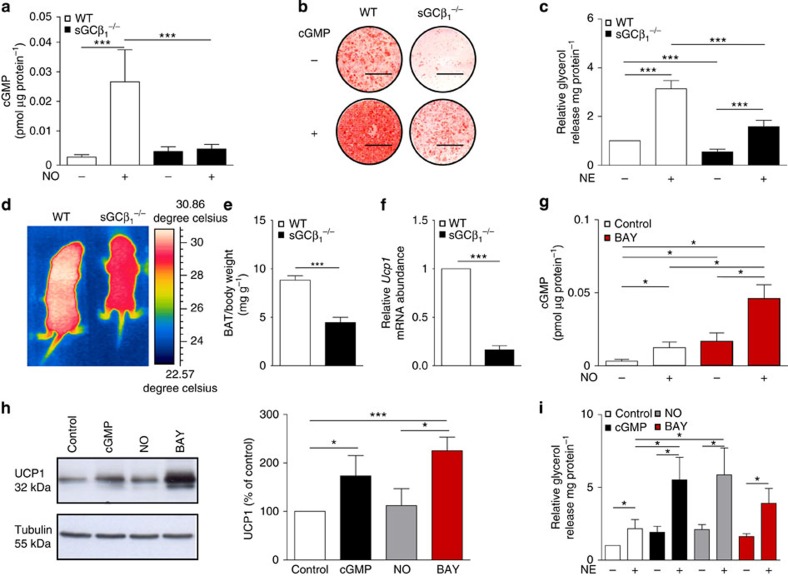
sGC is crucial for development and function of BAT. (**a**) Basal and NO-stimulated cGMP content of BA, *n*=5 independent cell cultures. (**b**) Oil Red-O stain of WT and sGCβ_1_^−/−^ BA differentiated in the presence or absence of 200 μM 8-pCPT-cGMP (cGMP), scale bar, 1 cm. (**c**) Lipolysis in WT and sGCβ_1_^−/−^ BA under basal and NE-stimulated conditions, *n*=5–7 independent cell cultures. (**d**) Representative thermographic image of newborn sGCβ_1_^−/−^ and WT mice. (**e**) Weight of BAT from newborn WT and sGCβ_1_^−/−^ mice, *n*=8 mice per genotype. (**f**) *Ucp1* gene expression in BAT of newborn mice, *n*=6 per genotype. (**g**) Basal NO- and BAY-stimulated intracellular cGMP levels of WT BA, which were incubated for 15 min with the indicated compounds, *n*=4 independent cell cultures. (**h**) UCP1 expression of WT BA differentiated in the presence of cGMP, 50 μM DETA/NO (NO) or 3μM BAY, representative western blot (left) and densitometric quantification normalized to loading control tubulin (right), *n*=3–4 independent cell cultures. (**i**) Lipolysis in BA under basal and NE-stimulated conditions, *n*=4–5 independent cell cultures. All data were assessed using Student's *t*-test and are presented as means±s.e.m. *P<0.05; ***P<0.005.

All three functional sGC subunits^26^ were detected in mature BA (Supplementary Fig. 1a). To address the physiological importance and whether sGC might be a potential drug target against obesity, transgenic mice lacking the β_1_ subunit of sGC (sGCβ_1_^−/−^)^27^, which contains the haeme/NO-binding domain, were analysed. Importantly, addition of NO increased cGMP only in wild-type (WT) cells, but not in sGCβ_1_^−/−^BA (Fig. 1a), showing that sGCβ_1_ is required for NO-dependent cGMP formation in BA. Differentiation of sGCβ_1_^−/−^ BA was diminished as indicated by reduced Oil Red-O staining (Fig. 1b) and reduced cellular triglyceride (TG) content (Supplementary Fig. 1b). Moreover, expression of adipogenic markers such as peroxisome proliferator-activated receptor gamma (PPARγ) and adipocyte protein 2 (aP2; Supplementary Fig. 1c,d), as well as of thermogenic markers such as UCP1, cytochrome *c* (Cytc), PPAR co-activator 1-alpha (PGC1α) and mitochondrial DNA content (Supplementary Fig. 1e–h) were significantly reduced in sGCβ_1_^−/−^ cells. cGMP rescued BA differentiation (Fig. 1b, Supplementary Fig. 1b–h). Basal and NE-induced lipolysis—important parameters for BA function—was significantly reduced by 46% and 51% in sGCβ_1_^−/−^ cells, respectively (Fig. 1c). The specificity of BAY in BA was confirmed in sGCβ_1_^−/−^ cells differentiated in the presence of BAY. In contrast to WT BA, BAY did not increase expression of the thermogenic markers UCP1, PGC1α and PPARγ (Supplementary Fig. 2a–c).

### sGC ablation results in dysfunctional thermogenesis *in vivo*

sGCβ_1_^−/−^ mice displayed reduced BAT-dependent thermogenesis and a significantly lower body surface temperature as analysed with infrared thermographic imaging^20^ in newborn mice (Fig. 1d, Supplementary Fig. 2d). Notably, sGCβ_1_^−/−^mice exhibited a significant reduction of BAT mass (−50%; Fig. 1e). *Ucp1* gene expression in BAT of sGCβ_1_^−/−^ mice was reduced to 16.5% of the WT level (Fig. 1f). Taken together, these data show that sGC is essential for BAT differentiation and function.

### BA function is increased after sGC stimulation

In order to stimulate sGC and recruit BAT pharmacologically, BAY 41-8543 (BAY) was used^28^. BAY (3 μM) significantly increased cGMP and acted synergistically with NO (Fig. 1g), which is a key feature of sGC stimulators^29^. Incubation of BA with BAY during differentiation enhanced adipogenic differentiation as demonstrated with Oil Red-O staining (Supplementary Fig. 3a) and increased intracellular TG content (Supplementary Fig. 3b). Furthermore, expression levels of markers for the adipogenic and thermogenic programme were increased (Fig. 1h and Supplementary Fig. 3c–e). Importantly, BAY-incubated cells showed increased basal and 1.8-fold higher NE-stimulated lipolysis (Fig. 1i). Together, these results show that BAY efficiently stimulates cGMP signalling in BA and recruits BA, resulting in enhanced function.

### sGC stimulation protects against DIO

**Figure 2:**
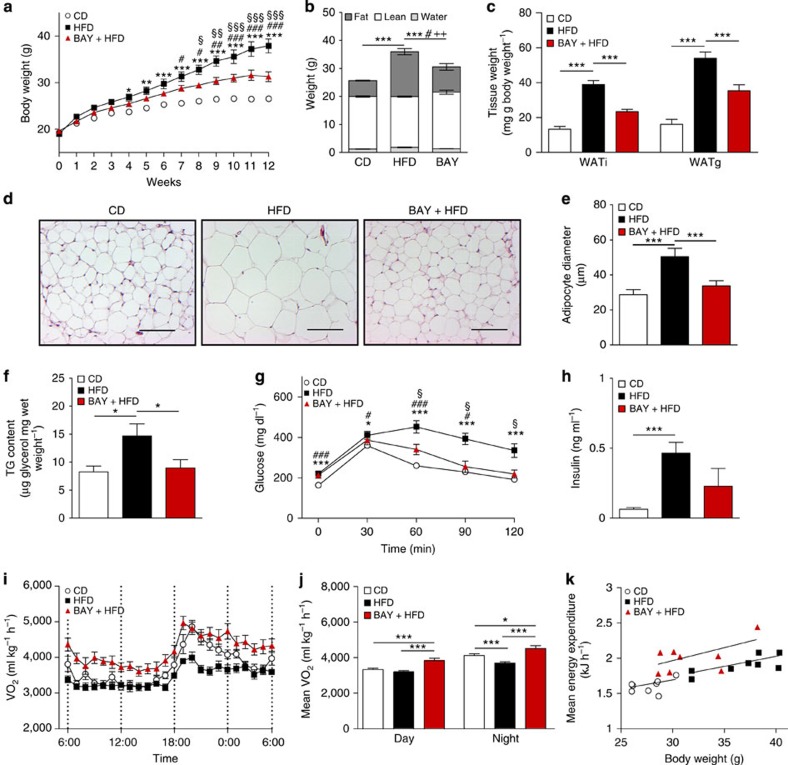
Stimulation of sGC reduces diet-induced obesity. (**a**,**b**) Effects of sGC stimulation on body weights (**a**) and body composition (**b**) of mice maintained on chow CD or HFD with or without BAY, *n*=8 mice per group. For (**a**) *HFD versus CD; #BAY versus CD; §BAY versus HFD and for (**b**) *fat; #lean;+free water. (**c**) Weights of WATi and gonadal WAT (WATg) deposits after treatment with or without BAY. (**d**,**e**) Representative images of WATi sections stained with haematoxylin and eosin, scale bar, 100 μm (**d**) and diameter of adipocytes in WATi (**e**). (**f**) TG content of the liver from mice fed CD or HFD with or without BAY. (**g**) Glucose tolerance test performed in week 12 of the study, *HFD versus CD; #BAY versus CD; §BAY versus HFD. (**h**) Insulin plasma levels at the end of the study. (**i**,**j**) Oxygen consumption (VO_2_) (**i**) and the mean oxygen consumption during day and night (**j**) of mice fed CD, HFD or HFD+BAY. (**k**) The mean energy expenditure in relation to body weight. All data were assessed using Student's *t*-test, except in **a**, where two-way analysis of variance (ANOVA) was applied and are presented as means±s.e.m. *^,§,#^*P*<0.05; **^, §§,##^*P*<0.01; ***^, §§§,###^*P*<0.005.

Next, the role of sGC in whole-body metabolism was studied. sGC stimulation protected mice from DIO, resulting in a 37% reduced body mass (Fig. 2a), with a 15% reduction in relative fat mass when compared with mice on high-fat diet (HFD) without BAY (Fig. 2b). Weights of inguinal (WATi) and gonadal WAT depots were 46% and 55% lower, respectively, in BAY-treated mice (Fig. 2c). Concomitantly, adipocyte size and hepatic lipid content were significantly decreased (Fig. 2d–f). BAY-treated mice exhibited improved glucose tolerance and a 50% reduction in plasma insulin levels (Fig. 2g,h). Indirect calorimetry revealed increased oxygen consumption in BAY-treated mice compared with chow control diet (CD) and HFD-fed mice (Fig. 2i,j), indicating a higher EE in BAY-treated mice. The difference in EE was most apparent when mice from all three groups with similar weights were compared (Fig. 2k). BAY-treated mice took up 1.8-fold more energy than mice fed HFD without BAY, but still remained leaner (Supplementary Fig. 4a). Locomotor activity was not significantly different between the groups (Supplementary Fig. 4b).

### Lipid uptake into BAT is increased by sGC stimulation

**Figure 3:**
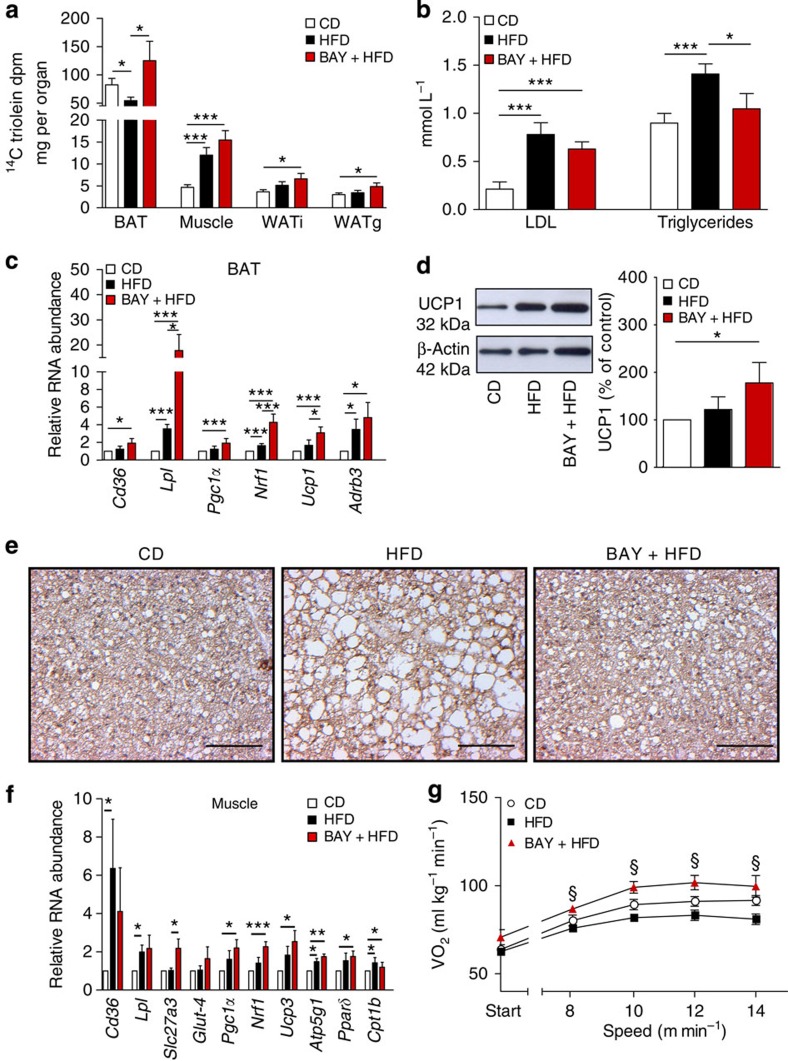
Stimulation of sGC increases lipid uptake into BAT. (**a**) Lipid uptake (^14^C triolein) into BAT, muscle, WATi and WAT g of mice fed chow CD or HFD with or without BAY. (**b**) Plasma LDL and triglyceride levels in CD, HFD or BAY-fed mice. (**c**) Expression of marker genes for lipid uptake (*Cd36* and *Lpl*), for mitochondrial biogenesis (*Pgc1*α and *Nrf1*) and thermogenesis (*Ucp1* and *Adrb3*) in BAT. (**d**) UCP1 protein expression in BAT, representative western blot (left) and densitometric quantification normalized to loading control tubulin (right). (**e**) Representative BAT sections stained for UCP1 and with haematoxylin, scale bar, 100 μm. (**f**) Gene expression of markers for lipid (*Cd36*, *Lpl* and *Slc27a3*), glucose uptake (*Glut-4*), mitochondrial biogenesis (*Pgc1*α and *Nrf1*), mitochondrial oxidative function (*Pgc1*α, *Ucp3* and *Atp5g1*) and fatty acid catabolism (*Pparδ* and *Cpt1b*) in the muscle. (**g**) Oxygen consumption (VO_2_) under exercise conditions, §BAY+HFD versus HFD. All data were assessed using Student's *t*-test and are presented as means±s.e.m. *^,§^*P*<0.05; **P<0.01; ***P<0.005. (**a**) *n*=6–8 mice per group, (**b**–**g**) *n*=8 mice per group.

BAT is a major sink for circulating TGs with the ability to reduce plasma lipid concentrations^30^. Lipid uptake into BAT of BAY-treated mice was 2.3-fold higher than in HFD-fed mice without BAY treatment (Fig. 3a). Although overall uptake of lipids into the muscle and WAT was much lower than in BAT, we observed a trend for higher lipid uptake in BAY-treated mice also into these tissues. Consistent with increased lipid shuttling into BAT, low density lipoprotein (LDL) and TG levels were decreased by 20% and 25%, respectively (Fig. 3b). Increased lipid uptake in BAT of BAY-treated mice was accompanied by significantly increased expression of genes involved in lipid uptake^30^ (*Cd36* and *Lpl*), as well as of genes important for mitochondrial biogenesis^31^,^32^ (*Pgc1α* and *Nrf1*; Fig. 3c). In addition, expression levels of thermogenic genes^4^ (*Ucp1* and *Adrb3*) as well as UCP1 protein were increased compared with control mice (Fig. 3c,d). BAY-treated mice showed reduced droplet size and increased *Vegf* expression in BAT, indicating the reversal of HFD-induced ‘whitening' (Fig. 3e, Supplementary Fig. 5a). Furthermore, NE-induced thermogenesis and mitochondrial DNA (mtDNA) content of BAT were significantly increased in BAY-treated mice (Supplementary Fig. 5b–e), demonstrating the increased thermogenic capacity of BAT after BAY treatment. In summary, these results indicate increased activity of BAT in BAY-treated mice resulting in enhanced clearance of nutrient lipids from the circulation.

### sGC stimulation induces browning

Enhanced lipid uptake into the muscle (Fig. 3a) was accompanied by increased expression of molecules involved in fatty acid^30^ (*Cd36*, *Lpl* and *Slc27a3*), glucose uptake (*Glut-4*) as well as of mitochondrial biogenesis^33^ (*Pgc1α* and *Nrf1*), mitochondrial oxidative function (*Ucp3* and *Atp5g1*) and fatty acid catabolism^34^ (*Pparδ* and *Cpt1b*; Fig. 3f). Analysis of oxygen consumption under exercise conditions revealed that BAY-treated mice had significantly higher oxygen consumption than untreated HFD-fed mice indicating higher EE by muscle tissue on BAY treatment during exercise (Fig. 3g).

**Figure 4:**
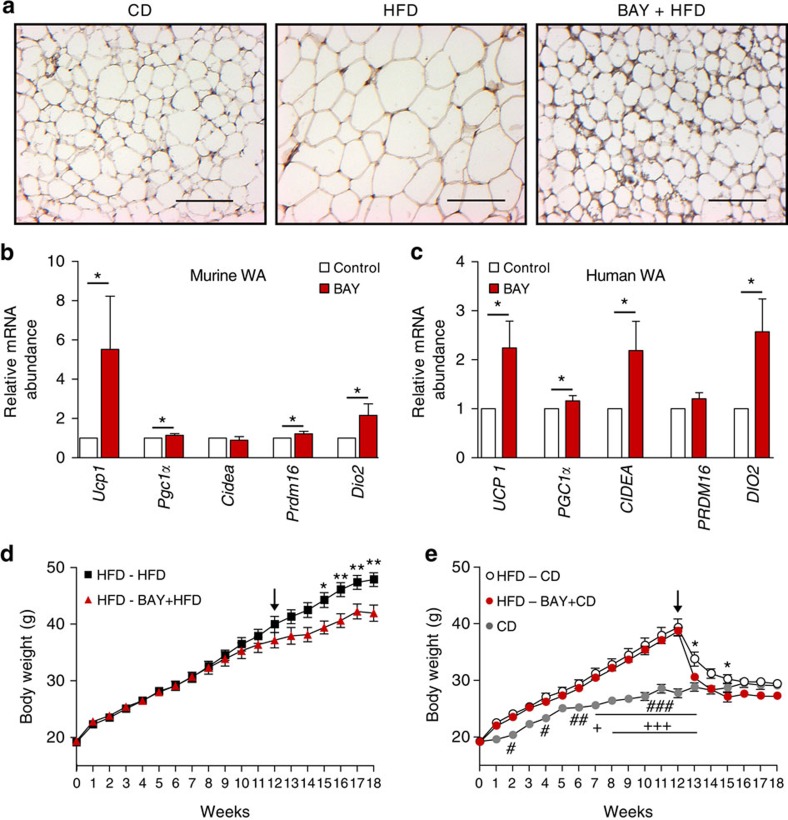
BAY induces browning and weight loss in already established obesity. (**a**) Representative WATi sections stained for UCP1 and with haematoxylin of mice that were fed chow CD, HFD or HFD+BAY. Scale bar, 100 μm. (**b**) Thermogenic marker gene expression in primary murine WA incubated with or without 3 μM BAY, *n*=5–7 independent cell cultures. (**c**) Abundance of thermogenic marker genes in human WA incubated with or without 3 μM BAY, *n*=6–8 independent cell cultures. (**d**) Body weights of mice that were fed a HFD for 12 weeks and then (arrow) fed a HFD with or without BAY for the six additional weeks, *n*=8 mice per group. (**e**) Body weights of mice that were fed a HFD for 12 weeks and then (arrow) switched CD with or without BAY for 6 weeks, *n*=8 mice per group, #CD versus HFD-CD,+CD versus HFD-BAY+CD, *HFD-CD versus HFD-BAY+CD. All data in **a**–**c** were assessed using Student's *t*-test, in **d**,**e** using two-way ANOVA and are presented as means±s.e.m. *^,#,+^*P*<0.05; **^, ##^*P*<0.01; ^###, +++^*P*<0.005.

As lipid uptake into WAT was increased in BAY-treated mice (Fig. 3a), we studied whether browning of WAT was induced by sGC stimulation. Histological analysis revealed UCP1-positive, multilocular cells (Fig. 4a). Moreover, BAY increased mitochondrial DNA content in WATi of BAY-treated mice (Supplementary Fig. 5d,e) and induced expression of the BA genes *Ucp1*, *Pgc1α*, *Prdm16* and *Dio2* and in murine WA (Fig. 4b). Since BAY stimulates browning of murine WA, we studied its effects in human adipocytes. Also in human WA, the sGC stimulator induced browning as indicated by significantly increased expression of the thermogenic markers *UCP1*, *PGC1α*, *CIDEA* and *DIO2* (Fig. 4c). Furthermore, incubation of a human BA differentiated from multipotent adipose-derived stem cells (hMADS)^25^,^35^ with BAY significantly increased expression of thermogenic markers compared with unstimulated control (Supplementary Fig. 5f). Together, these results imply that BAY could have the potential to induce or enhance the BA thermogenic programme in human WA and BA.

### sGC stimulation induces weight loss in established obesity

To assess whether sGC stimulation can be used to induce weight loss in a clinically more relevant setting, that is, already established obesity, mice were fed a HFD for 12 weeks to induce DIO, followed by treatment of HFD with BAY for additional 6 weeks (Fig. 4d). Obese mice receiving BAY were 12% lighter than mice receiving HFD without BAY. This was accompanied by a 37% reduction in WATi mass and a significant improvement of glucose clearance (Supplementary Fig. 6a,b). Oxygen consumption of BAY-treated mice fed a HFD was significantly higher during light and dark phases compared with control mice on HFD (Supplementary Fig. 6c).

Finally, we switched obese mice to normal CD with and without BAY to mimic a conventional weight loss scheme on the basis of calorie restriction. The switch to CD-normalized body weight and BAY treatment enhanced weight loss resulting in a further 7% decrease of body weight (Fig. 4e). BAY treatment during calorie reduction (BAY+CD) decreased WATi mass by 23% compared with mice receiving CD alone (Supplementary Fig. 6d). Again, glucose clearance was significantly improved by sGC stimulation (Supplementary Fig. 6e). In line, BAY also increased oxygen consumption of mice switched to CD after 12 weeks of HFD compared with control mice (Supplementary Fig. 6f). Together, these data show that sGC stimulation can be used to enhance EE in already established obesity leading to a significant weight reduction even during a continuous high-calorie diet.

## Discussion

The results of our study show that pharmacological sGC stimulation protects against DIO and induces weight loss in already established obesity. The changes in body weight induced by the sGC stimulator are accompanied by improved overall metabolic status as indicated by improved glucose tolerance, decreased liver steatosis, reduced insulin levels and decreased adipocyte size in WATi, a parameter for healthy expansion of WAT^36^. Importantly, EE was increased under basal conditions and we found significantly increased lipid uptake into BAT. Previously, it has been shown that active BAT can take up ∼50% of nutrient lipids^30^,^37^ resulting in increased EE^19^,^38^. Moreover, also muscle and WAT tended to take up more lipids after treatment with the sGC stimulator and, therefore, could contribute to the increase in EE.

In this context, it is of interest that EE was increased during exercise after sGC stimulation and that mitochondrial biogenesis was increased in muscle of BAY-treated mice. Similar results were observed in a genetic model of increased downstream cGMP signalling where mitochondrial biogenesis and EE were increased in mice overexpressing the cGMP-target PKGI^34^. These data indicate that sGC stimulators might be used to enhance weight loss induced by physical activity.

Mice treated with the sGC stimulator showed ‘browning' of WAT, that is, the appearance of inducible BA in WAT, which is in line with previous studies^23^,^25^ that have shown cGMP-dependent browning of WAT. In addition, we found that HFD-induced ‘whitening' of classical, interscapular BAT was reduced on sGC stimulation. Thus, sGC stimulation recruits BAT and increases EE with increased uptake of lipids especially into BAT. Our data might also extend to humans as sGC stimulation induced thermogenic markers in human WA and BA.

The downstream cGMP pathway is an important regulator of BAT that mediates the effects of BAY and has been deciphered earlier^9^. It consists of the cGMP-producing enzymes sGC and particulate guanylyl cyclases (NPR-A and NPR-B). PGKI, the major downstream target of sGC, is indispensible for thermogenic differentiation of BA *in vitro* and *in vivo* and is crucially involved in browning of WAT^20^,^23^. PKGI inhibits RhoA that results in the release of the RhoA/ROCK-dependent inhibition of the insulin/IRS-1/PI3K/Akt pathway. In the activated state, the cGMP pathway results in mitochondrial biogenesis and induction of UCP1 (refs 20, 21). On the other hand, phosphodiesterases (PDEs) reduce the levels of cGMP in adipocytes^39^ and inhibition of PDE5 results in browning^23^.

Here we focused on sGC as a new target in antiobesity therapy to modulate cGMP signalling at the level of cGMP production. Mice lacking the β_1_-subunit of sGC, which contains the haeme-binding domain that is critical for activation of sGC by NO and BAY^40^,^41^, show a severe phenotype with reduced BAT-derived thermogenesis and altered differentiation of BA. Thus, sGC is essential for normal differentiation of BAT.

This important signalling cascade can be regulated pharmacologically at several levels. Organic nitrates that deliver NO have been used to treat angina pectoris for more than 100 years^42^, and NO has been shown to be involved in mitochondrial biogenesis in a broad range of cells including BA^21^. Its volatile nature and primary action in the vasculature argue against a BAT-specific effect of NO in a possible therapeutic application of NO in obesity. Furthermore, nitrate tolerance^43^ is a major drawback that develops rapidly making long-term application not applicable, which would be necessary in antiobesity therapies.

Natriuretic peptides that activate NPR-A and NPR-B have been shown to increase thermogenic markers in human BA, are capable of increasing EE in mice with concomitant browning of WAT^25^ and have been shown to counteract DIO^34^. In light of the positive effects of natriuretic peptides on EE^25^, it is of special interest that ablation of sGCβ_1_ has such a profound effect on BA differentiation and function demonstrating the central role of sGC-derived cGMP in BAT. Brain natriuretic peptide (BNP) has been used pharmacologically to treat acute decompensated heart failure; however, its safety has been questioned^44^,^45^ and the use of BNP might not be feasible in all obesity patients.

Another way to pharmacologically modulate cGMP signalling is the use of PDE5 inhibitors such as sildenafil, which leads to increased cGMP levels. Members of this drug class have been used mainly for the treatment of erectile dysfunction for several years^46^. In mice, short-term treatment with sildenafil results in browning of WAT^23^ and long-term treatment with sildenafil induced weight reduction in mice on HFD^47^. Important side effects of these drugs including interactions with agents that lower blood pressure such as nitrates^48^ or alpha blockers^49^ and an increased risk for development of melanoma^50^ have been reported.

Thus, alternative strategies for enhancing cGMP signalling in adipocytes at different levels of the cascade are required for rational therapy. Modulating signalling pathways at different levels has been proven to be a prerequisite for successful treatment of multilayered diseases in heterogeneous groups of patients. For example, the development of several pharmacological strategies to regulate the renin–angiotensin–aldosterone system resulted in different classes of drugs that can be administered to different subpopulations of patients with hypertension and renal disorders as well as cardiovascular disease and provide efficient alternative treatment options.

To increase sGC-dependent cGMP production, we used a member of the new drug class of sGC stimulators. These compounds stimulate the native sGC in a haeme-dependent manner and show a strong synergism with NO (ref. 29). These characteristics allow sGC stimulators to increase cGMP signalling even when endogenous NO/cGMP signalling is impaired because of reduced bioavailability of NO. The compound used in this study is chemically closely related to riociguat^51^, which is used for the treatment of pulmonary hypertension and shows a favourable safety profile^52^.

In the clinical trials with the sGC stimulator riociguat, the included patients had normal body weights^52^,^53^. Importantly, patients with obesity were excluded from the studies. Moreover, patients suffering from pulmonary hypertension rather tend to have a lower body because of their severe disease. A significant change in body weight of the patients who were included was not observed in the clinical trials with riociguat.

In the light of the results from the Collins laboratory, which show that natriuretic peptides increase EE and induce browning in animal models, the concept of natriuretic peptides as antiobesity drugs seems to be promising^25^. To our knowledge, an effect of administered natriuretic peptides on body weight in clinical trials has not been published so far. Clinical trials investigating the effects of natriuretic peptides on obesity are underway^54^.

In summary, pharmacological stimulation of sGC increases the function of BA and induces a brown, energy-combusting phenotype in WA. Importantly, sGC stimulation counteracts DIO-induced pathologies even in already established obesity via increased energy utilization. Activation of sGC results in increased lipid uptake and usage mainly by BAT. Overall, sGC stimulation leads to reduced body weight and an improved metabolic phenotype in mice with DIO. sGC stimulation represents an innovative pharmacological principle and sGC stimulators are potential candidates for the treatment of obesity and associated comorbidities.

## Methods

### Materials

Antibodies against PPARγ and aP2 were purchased from Santa Cruz Biotechnology, CytC from BD Bioscience, UCP1, β-Actin and sGCα_1_ from Sigma, tubulin from Dianova, and sGCβ_1_ and sGCα_2_ from Cayman and Abcam, respectively. BAY 41–8543 (BAY) was provided by Bayer HealthCare AG.

### Primary human and murine adipocyte culture

Stromal vascular fraction from murine intrascapular BAT was isolated and differentiated as described previously^20^. In brief, preadipocytes and mesenchymal stem cells within the stromal vascular fraction were isolated from the surrounding tissue by collagenase digestion and immortalized using SV40 large T antigen lentivirus. The cells were seeded in DMEM containing 10% fetal bovine serum (FBS) and 100 IU penicillin, 100 μg ml^−1^streptomycin (P/S). The medium was exchanged to differentiation medium (DM) containing 20 nM insulin and 1 nM tri-iodothyronine (T3) every other day. Differentiation was induced by addition of 0.5 mM isobutylmethylxynthine (IBMX) and 1 μM dexamethasone to DM 4 days after seeding. hMADS were kindly provided under MTA by C. Dani (University of Nice Sophia Antipolis) and were grown in DMEM supplemented with 2 mM glutamine, 10 mM Hepes buffer, 1% P/S and 2.5 ng ml^−1^ FGF2. To induce differentiation, the medium was changed to DMEM/Ham's F12 (50/50) containing 5 μg ml^−1^ insulin, 10 μg ml^−1^ transferrin, 0.2 nM T3, 1 μM rosiglitazone, 100 μM IBMX and 1 μM dexamthasone. After 3 days, the medium was changed to medium without IBMX and dexamethasone that was exchanged every other day^35^. Human WA were obtained from Promo-Cell and differentiated according to the manufacturer's protocols using commercially available premixed media. Murine WA were differentiated as described^55^. Briefly, preadipocytes were isolated from WATi of 8-week-old C57Bl/6 mice and grown in DMEM containing 10% FBS and 1% P/S, and differentiation was induced using DMEM containing 5% FBS, 1% P/S, 0.172 μM insulin, 1 nM T3, 1 μM rosiglitazone, 50 μg ml^−1^ L-ascorbate, 1 μM D-biotin, 17 μM panthothenat, 0.5 mM IBMX and 0.25 μM dexamethasone. After 2 days, the medium was changed to a medium without rosiglitazone, IBMX and dexamethasone and replenished every other day. Cells were incubated with or without 200 μM 8-pCPT-cGMP (Biolog), 3 μM BAY or 50 μM DETA/NO (Sigma) as indicated. Thermogenic marker gene expression was analysed after 8-h stimulation of mature cells.

Oil Red-O staining was achieved in paraformaldehyde-fixed cells by incubating the cells in 3 mg ml^−1^ Oil Red-O in isopropyl alcohol. Lipolysis and intracellular TGs were measured using the commercially available Free Glycerol Determination Kit (Sigma)^55^. NE (10 nM) was used to maximally induce lipolysis. cGMP levels were determined using EIA (enzyme immonoassay) (Cayman Chemical) according to the manufacturer's instructions.

### Western blot and quantification

Protein amount was quantified using the Bradford assay and concentration was normalized before western blot analysis. Western blots were carried out using the standard procedures. Band intensity was quantified using the Quantity One software (Bio-Rad). Background was subtracted from all data that were normalized to loading controls. Following antibodies were used: against UCP1 from Sigma diluted 1:500, against PPARγ from Santa Cruz Biotechnology diluted 1:1,000, against sGCα_1_ from Sigma diluted 1:500, against sGCα_2_ from Abcam diluted 1:250, against sGCβ_1_ from Cayman diluted 1:1,000, against aP2 from Santa Cruz diluted 1:1,000, against Cytc from Santa Cruz diluted 1:1,000, against tubulin from Dianova diluted 1:1,000, against β-Actin from Sigma diluted 1:1,000. Full blots of blot sections are shown in Supplementary Fig. 7.

### RNA isolation and gene expression analysis using qRT–PCR

Total RNA was extracted from tissue or cells using InnuSolv (AnalytikJena). RNA (500 ng) was reverse-transcribed with the Transcriptor First Strand synthesis kit (Roche) using random hexamer primers^20^. qRT–PCR was performed with SYBR-Green (Roche, Life Technologies) on a HT7900 instrument (Applied Biosystems) or ViiA7 instrument (Applied Biosystems). Using relative quantification methods, fold changes were calculated with human glyceraldehyde 3-phosphate deydrogenase or murine hypoxanthine guanine phosphoribosyltransferase as internal controls. Primer sequences are listed in Supplementary Tables 1 and 2.

### Analysis of mitochondrial DNA

Genomic DNA was isolated and qRT–PCR was performed. The amount of mtDNA was normalized to the amount of chromosomal (H19) DNA^20^. Primer sequences are listed in Supplementary Table 3.

### Animal studies

Six-week-old male C57Bl/6J mice were purchased from Charles River. HFD (60% of calories from fat, D12492) and chow CD (D12450B) were purchased from Ssniff GmbH, Germany. BAY (300 mg kg^−1^) were directly added to the diet as indicated. Mice were maintained on a daily cycle of 12 h light (0600–1,800) and 12 h darkness (1,800–0600), at 24±1 °C, and were allowed free access to diets and water.

During the studies mice were weighed weekly. All animal studies were conducted according to the German animal welfare law and permitted by the Landesamt für Natur, Umwelt und Verbraucherschutz (LANUV) Nordrhein-Westfalen, Germany.

### Thermography

Infrared thermography on newborn mice was performed at ambient temperature using a hand-held infrared camera (IC060, Trotec GmbH), and images were analysed using the IC-Report software 1.2 (Trotec GmbH)^20^.

### Body composition

Body composition in conscious mice was determined with the use of a benchtop NMR device Minispec (Bruker Corporation).

### Immunohistochemistry

Five-micrometre paraffin-embedded BAT and WAT sections were incubated with an antibody against UCP1 (Sigma), diluted at 1:50, at 4 °C overnight followed by 1 h incubation with the secondary antibody (SignalStain Boost IHC, Cell Signaling) at room temperature and visualization using a commercially available DAB kit (Vector Laboratories)^19^. Haematoxylin was used for counterstaining. Haematoxylin and eosin staining was performed on 5-μm paraffin-embedded WATi sections following the standard procedures. Adipocyte diameter was measured using ImageJ.

### Glucose tolerance test

Animals were fasted for 5 h before intraperitoneal injection of 8 μl g^−1^ body weight of a glucose solution (0.25 g ml^−1^). Glucose was measured before and 30, 60, 90 and 120 min post injection in blood drawn from the tail vein using dip sticks (Roche)^19^.

### Indirect calorimetry

Individual oxygen consumption (VO_2_) and CO_2_ production (VCO_2_) were measured using a Phenomaster device (TSE systems) at ambient temperature^56^. During measurement, mice had *ad libitum* access to food and water. The resting metabolic rate was measured at 30 °C, and NE-induced respiration was measured at 23 °C after subcutaneous injection of 1 mg kg^−1^ NE (Arterenol, Sanofi). Exercise conditions were induced by increasing speed by 2 m min^−1^, starting at a velocity of 8 m min^−1^. EE was calculated using the following equation: EE (kJ h^−1^)={4.44+1.43 × (VO_2_/VCO_2_)} × VO_2_ (ml O_2 _h^−1^) × 360.

### Plasma parameters

Insulin was measured using a commercially available ELISA following the manufacturer's instructions (Crystal Chem). LDL and TGs were measured using a Cobas device (Roche).

### TG levels *in vivo*

TGs were measured in minced liver samples using the Free Glycerol Determination Kit (Sigma) and normalized to wet weight.

### Lipid uptake

Mice were fed CD, HFD or HFD+BAY for 8 weeks and intraperitoneally injected with 1 mg kg^−1^ BAY or vehicle to guarantee high plasma levels. Lipid uptake into tissues was performed as described previously^30^. In brief, radiolabelled triolein was administered by oral gavage. Mice were killed 2 h post injection and organs were harvested. Radioactivity was counted in solubilized organs by scintillation counting.

### Statistics

Two-tailed Student's *t*-tests or analysis of variance with Bonferroni *post hoc* tests for multiple comparisons were used where appropriate. *P* values below 0.05 were considered significant. Statistical analysis was performed with the GraphPad prism 5 software. All data are represented as mean±s.e.m. The sample size was chosen based on our previous metabolic studies^19^,^55^.

## Additional information

**How to cite this article:** Hoffmann, L. S. *et al.* Stimulation of soluble guanylyl cyclase protects against obesity by recruiting brown adipose tissue. *Nat. Commun.* 6:7235 doi: 10.1038/ncomms8235 (2015).

## Supplementary Material

Supplementary Information Supplementary Figures 1-7, Supplementary Tables 1-3 and Supplementary References
