# Supplementary material for: Stimulation of soluble guanylyl cyclase protects against obesity by recruiting brown adipose tissue
Source: Nat Commun. 2015 May 26;6:7235. doi: 10.1038/ncomms8235 (PMC4455111; doi:10.1038/ncomms8235)
Supplement: Supplementary Information — Supplementary Figures 1-7, Supplementary Tables 1-3 and Supplementary References [file ncomms8235-s1.pdf]

Supplementary Figure 1

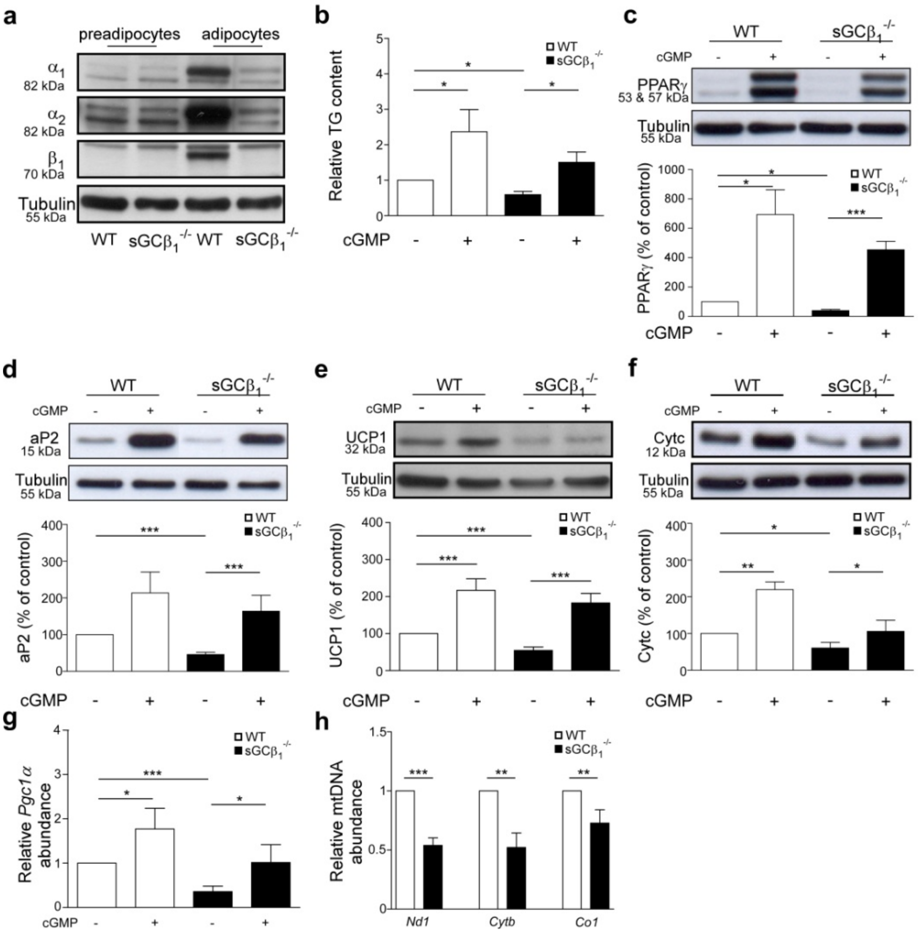

Supplementary Figure 1 │ sGC is central for brown adipocyte differentiation and function. (**a**) sGC subunit expression in WT and sGC$\beta_1^{-/-}$ preadipocytes and mature BA determined by Western blot. (**b**) Intracellular TG content in sGC$\beta_1^{-/-}$ and WT cells differentiated in the presence or absence of cGMP, n=5 independent cell cultures. (**c** – **f**) Protein expression levels of PPARγ (**c**), aP2 (**d**), UCP1 (**e**) and Cytc (**f**) in sGC$\beta_1^{-/-}$ and WT BA. Representative Western blots above and densitometric measurement below normalized to the loading control tubulin, n=4-8 independent cell cultures. (**g**) Gene expression levels of *Pgc1α* in WT and sGC$\beta_1^{-/-}$ BA, n=7-8 independent cell cultures. (**h**) Abundance of the mitochondrial DNA markers *NADH dehydrogenase* (*Nd1*), *cytochrome b* (*Cytb*) and *cytochrome c oxidase subunit 1* (*Co1*), n=3-5 independent cell cultures. All data were assessed using Student's *t*-test and are presented as means ± s.e.m. *P<0.05; **P<0.01; ***P<0.005.

Supplementary Figure 2

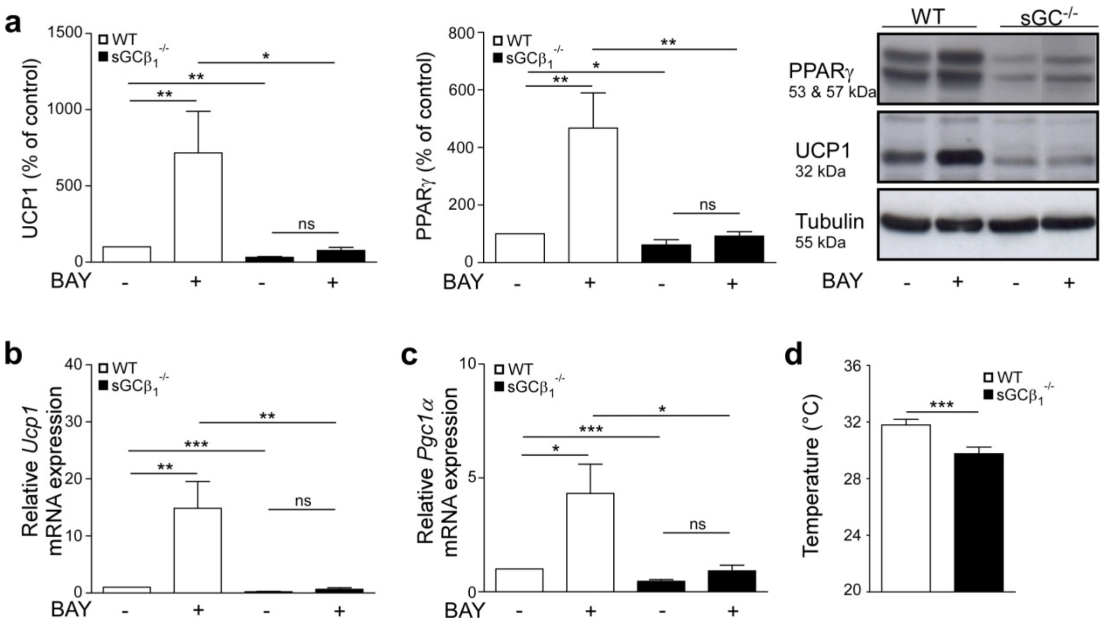

Supplementary Figure 2 │ Specificity of BAY on sGC in BA. SVF from BAT of sGC$\beta_1^{-/-}$ and WT mice were differentiated in the presence and absence of 3 µM BAY. (**a**) Protein expression of UCP1 and PPARγ in WT and sGC$\beta_1^{-/-}$ BA differentiated in the presence or absence of BAY. Representative Western blots (right) and densitometric quantification normalized to tubulin (left), n=6-7 independent cell cultures. (**b, c**) Influence of BAY on *Ucp1* (**b**) and *Pgc1α* (**c**) gene expression in WT and sGC$\beta_1^{-/-}$ BA differentiated with or without addition of BAY, n=6 independent cell cultures. (**d**) Interscapular temperature of sGC$\beta_1^{-/-}$ and WT newborn mice measured with infrared thermography, n=8-9 mice per genotype. All data were assessed using Student's *t*-test and are presented as means ± s.e.m., *P>0.05, **P<0.01.

Supplementary Figure 3

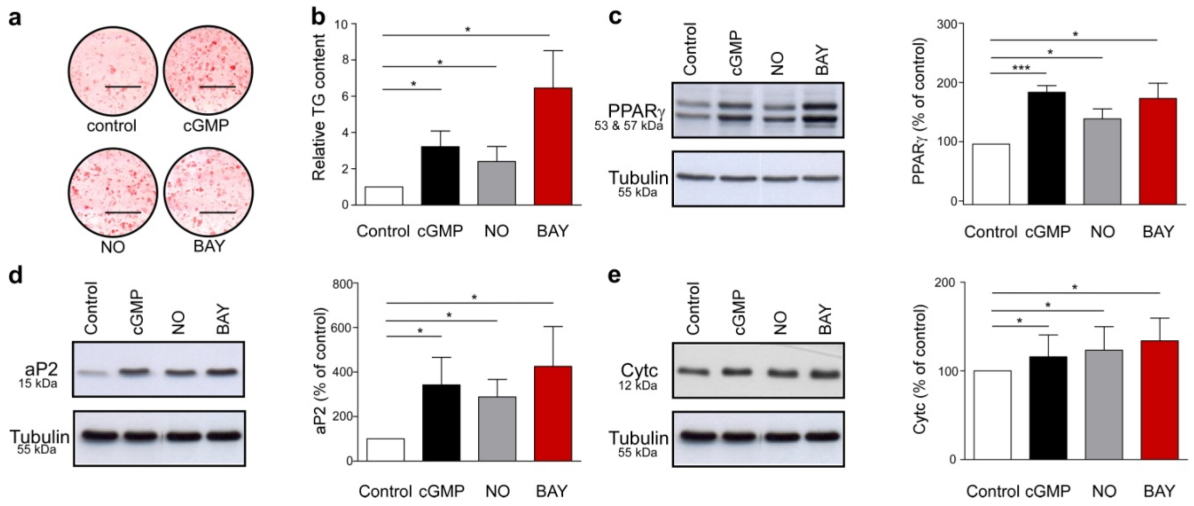

Supplementary Figure 3 │ sGC stimulation increases the adipogenic and thermogenic program in BA. (**a, b**) Representative Oil RedO stain, scale bar represents 1 cm, (**a**) and triglyceride content (**b**) of WT BA differentiated in the presence or absence of BAY, NO or cGMP, n=4 independent cell cultures (**b**). (**c - e**) Protein expression levels of PPARγ (**c**), aP2 (**d**) and Cytc (**e**) in BA. Representative Western blots on the left and densitometric measurement on the right normalized to the loading control tubulin, n=4 independent cell cultures. All data were assessed using Student's *t*-test and are presented as means ± s.e.m. *P<0.05; ***P<0.005.

Supplementary Figure 4

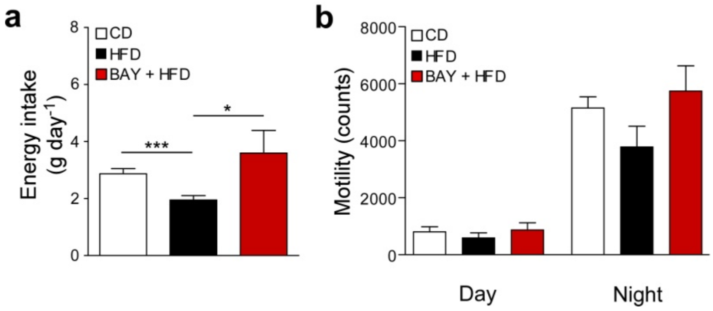

Supplementary Figure 4 │ Metabolic data of BAY-treated mice on a HFD. (**a**, **b**) Energy intake of mice within 24 h (**a**) and motility during light and dark phase (**b**) of mice fed control diet (CD) or high fat diet (HFD) with or without BAY, n=8 mice per group. All data were assessed using Student's *t*-test and are presented as means ± s.e.m. *P<0.05; ***P<0.005.

Supplementary Figure 5

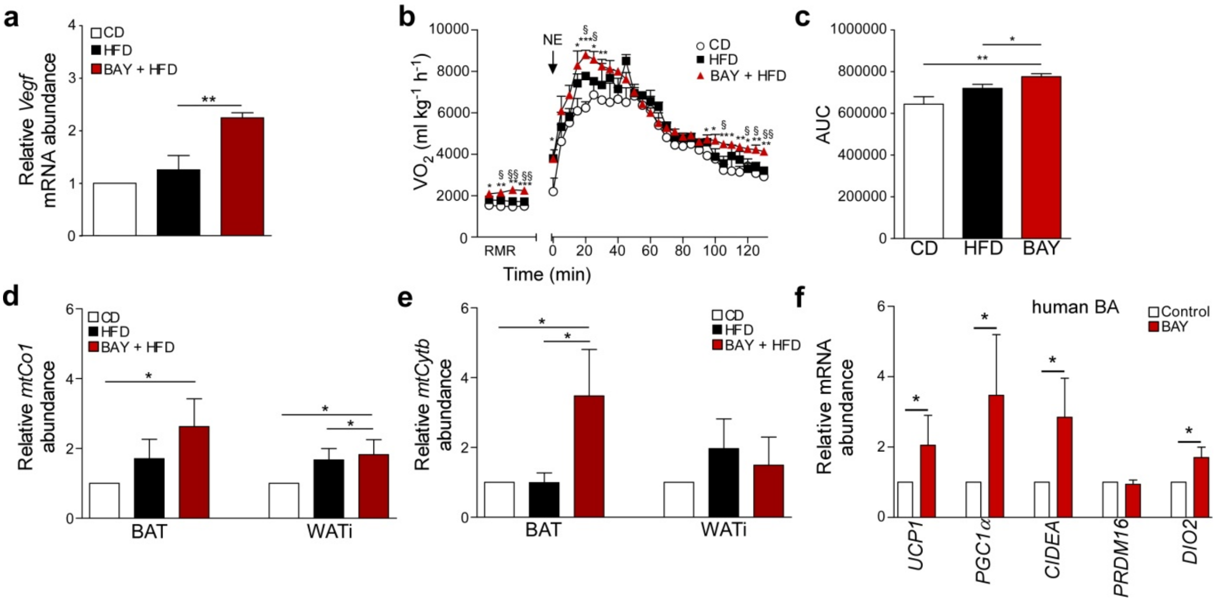

Supplementary Figure 5 │ sGC stimulation reduces whitening in BAT, increases Norepinephrine-induced thermogenesis and induces thermogenic markers in human BA. (**a**) *Vegf* expression in BAT of mice fed control diet (CD) or high fat diet (HFD) with or without addition of BAY, n=8 mice per group. (**b**) Norepinephrine-induced thermogenesis in BAY-treated mice. Mice were fed a CD, HFD or HFD+BAY (BAY) for 12 weeks. The resting metabolic rate was analysed at 30°C. Norepinephrine (NE; subcutaneous injection, 1 mg kg^{-1} body weight) was used to induce maximal thermogenesis. (**c**) Area under the curve (AUC) of mean oxygen consumption after NE injection, n=3 mice per group. (**d, e**) Mitochondrial DNA content in BAT and WATi. Mice were fed CD, HFD or HFD+BAY (BAY) for 12 weeks. Mitochondrial DNA encoded gene expression of *Cytochrome c oxidase subunit 1* (*mtCo1*) (**d**) and *Cytochrome b* (*mtCytb*) (**e**) were assessed by qRT-PCR and normalized to the chromosomal DNA encoded gene H19, n=5-7 per group. (**f**) Thermogenic marker gene expression in human BA differentiated from hMADS incubated with or without 3 µM BAY, n=4 independent cell cultures. All data were assessed using Student's *t*-test and are presented as means ± s.e.m. *$P<0.05$; **$P<0.01$.

Supplementary Figure 6

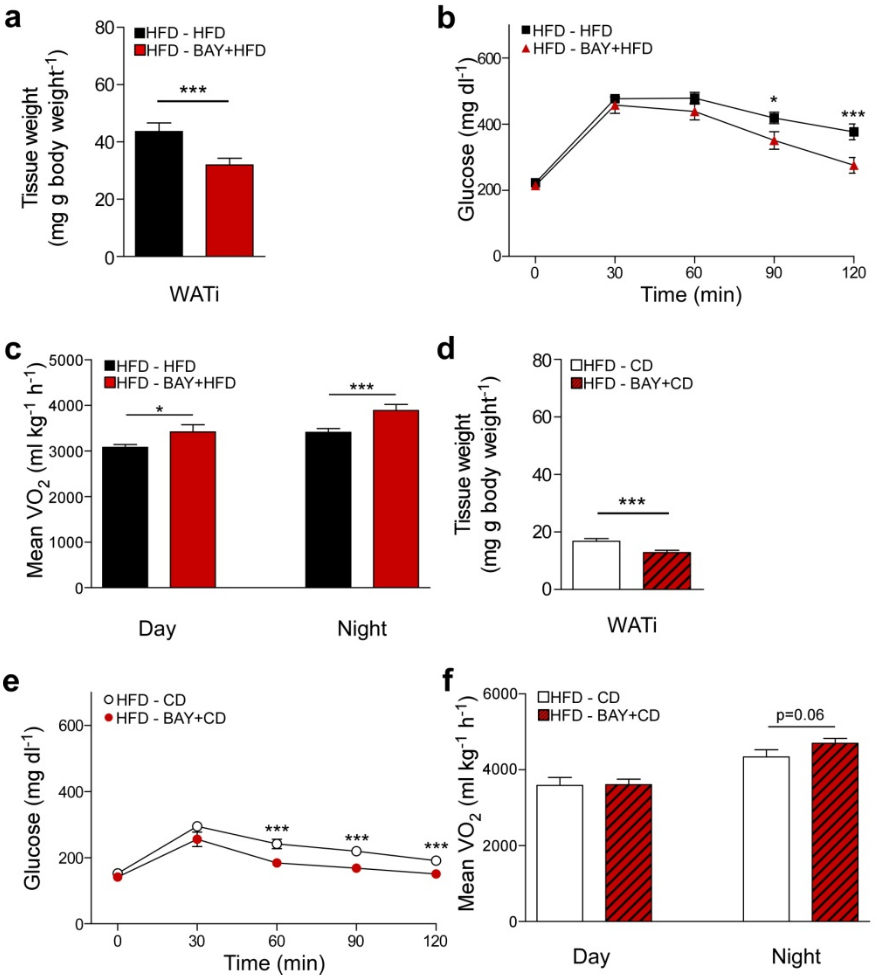

Supplementary Figure 6 │ sGC stimulation decreases WAT, improves glucose clearance and increases EE in established obesity. (**a**) Weights of inguinal WAT (WATi) depots of mice fed a high fat diet (HFD) for 12 weeks and treated with or without BAY for 6 additional weeks, n=8 mice per group. (**b**, **c**) Glucose tolerance test (**b**) and mean oxygen consumption (VO_2) (**c**). (**d**) Weights of WATi depots of mice fed a HFD for 12 weeks and then switched to control diet (CD) with or without BAY for 6 weeks, n=8 mice per group. (**e**, **f**) Glucose tolerance test (**e**) and mean oxygen consumption (VO_2) (**d**). All data were assessed using Student's *t*-test and are presented as means ± s.e.m. **P*<0.05; ****P*<0.005.

Supplementary Figure 7

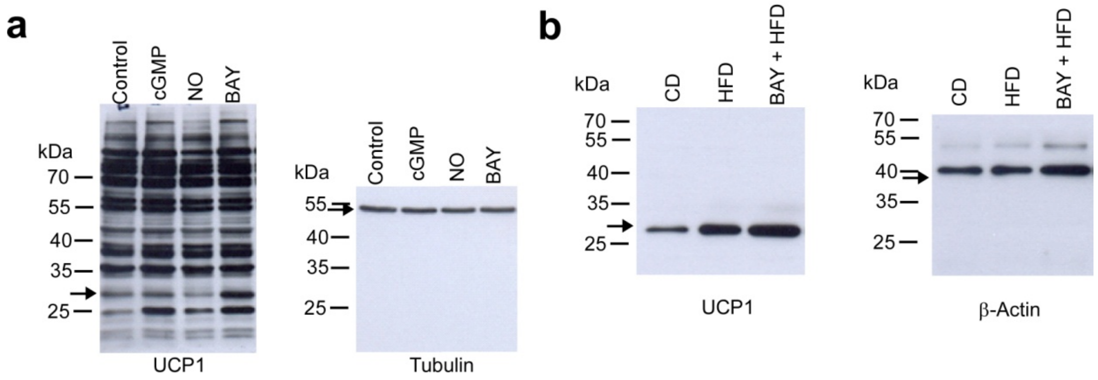

Supplementary Figure 7 │ Full blots of blot sections in the main paper. (**a**) Full blots of the sections shown in Figure 1h. (**b**) Full blots of the sections shown in Figure 3d.

Supplementary Tables

Supplementary Table 1: Primer sequences used to detect gene expression levels in murine samples

Gene	Forward	Reverse
Atp5g1	AGTTGGTGTGGCTGGATCA	GCTGCTTGAGAGATGGGTTC[1]
Adrb3	ATCTTCTCTCTGTGCTGGCTGCCCT	CATCGGTTCTGGAGCGTTGGAGAGT
Cd36	TGGCCAAGCTATTGCGACAT	AGGCATTGGCTGGAAGAACA
Cpt1b	GGCACCTCTTCTGCCTTTAC	TTTGGGTCAAACATGCAGAT[2]
Dio2	GCGATGGCAAAGATAGGTGA	GAATGGAGCTGGGTGTAGCA[3]
Glut-4	GACGACGGACACTCCATCTG	AGCTCTGCCACAATGAACCA
Hprt	ACATTGTGGCCCTCTGTGTGCTCA	CTGGCAACATCAACAGGACTCCTCGT[4]
Lpl	AGCAGCAAGACCTTCGTGG	TCTCTCTTGTACAGGGCGGC
Nrf1	TGTGGCAACAGGGAAGAAACGGAA	TCCGTAATGCCTGGGTCCATGAAA
Pgc1α	GCACACACCGCAATTCTCCCTTGTA	ACGCTGTCCCATGAGGTATTGACCA[4]
Pparδ	ACTGCAGCCCCCTATAGT	GGATCAGTTGGGTCAGTGGG
Slc27a3	TGGATTTGGTTCGGACTGGC	CTGGCTCATCCACTTGGTCT
Ucp1	TAAGCCGGCTGAGATCTTGT	GGCCTCTACGACTCAGTCCA[5]
Vegf	GGAGATCCTTCGAGGAGCACTT	GGCGATTTAGCAGCAGATATAAGAA[6]

Supplementary Table 2: Primer sequences used to detect gene expression levels in human samples

Gene	Forward	Reverse
CIDEA	GGCAGGTTCACGTGTGGATA	GAAACACAGTGTTTGGCTCAAGA[7]
DIO2	GTCACTGGTCAGCGTGGTTTT	TTCTTCACATCCCCCAATCCT[7]
GAPDH	TGGTCTCCTCTGACTTCA	GTGAGGGTCTCTCTCTTTCCT[5]
PGC1α	CTGTGTCACCACCCAAATCCTTAT	TGTGTCGAGAAAAGGACCTTGA[7]
PPARγ	AGCCTCATGAAGAGCCTTCCA	TCCGGAAGAAACCCTTGCA[7]
PRDM16	GAAACTTTATTGCCAATAGTGAGATGA	CCGTCCACGATCTGCATGT[7]
UCP1	GGAACAATCACCGCTGTGGT	ATCCTGAGAGAGGCGCAGCT[7]

Supplementary Table 3: Primer sequences used to detect mitochondrial DNA marker abundance

DNA marker	Forward	Reverse
Nd1	AATCGCCATAGCCTTCCTAACAT	GCCGTCTGCAAATGGTTGTAA[4]
Cytb	TTCTGAGGTGCCACAGTTATT	GAAGGAAAGGTATTAGGGCTAAA[4]
Co1	CCCAATCTCTACCAGCATC	GGCTCATAGTATAGCTAGGAG[4]
H19	GTACCCACCTGTCGTCC	GTCCACGAGACCAATGACTG[4]

Supplementary References

1 Yoon, J.C. *et al.*, Wnt signaling regulates mitochondrial physiology and insulin sensitivity. *Genes Dev.* **24,** 1507-1518 (2010).

2 Haemmerle, G. *et al.*, Atgl-mediated fat catabolism regulates cardiac mitochondrial function via ppar-alpha and pgc-1. *Nat. Med.* **17,** 1076-1085 (2011).

3 Seale, P. *et al.*, Transcriptional control of brown fat determination by prdm16. *Cell Metab.* **6,** 38-54 (2007).

4 Haas, B. *et al.*, Protein kinase g controls brown fat cell differentiation and mitochondrial biogenesis. *Sci. Signal.* **2,** ra78 (2009).

5 Bordicchia, M. *et al.*, Cardiac natriuretic peptides act via p38 mapk to induce the brown fat thermogenic program in mouse and human adipocytes. *J. Clin. Invest.* **122,** 1022-1036 (2012).

6 Shih, S.C. *et al.*, Molecular profiling of angiogenesis markers. *Am. J. Pathol.* **161,** 35-41 (2002).

7 Pisani, D.F. *et al.*, Differentiation of human adipose-derived stem cells into "Brite" (brown-in-white) adipocytes. *Front. Endocrinol. (Lausanne)* **2:87,** 10.3389/fendo.2011.00087 (2011).
